# Surgical Preparedness Index in Orthopaedics During the Coronavirus Disease 2019 (COVID-19) Pandemic

**DOI:** 10.7759/cureus.56066

**Published:** 2024-03-12

**Authors:** Madhan Jeyaraman, Naveen Jeyaraman, Karthikeyan P Iyengar, Sankalp Yadav

**Affiliations:** 1 Clinical Research, Virginia Tech India, Dr MGR Educational and Research Institute, Chennai, IND; 2 Orthopaedics, ACS Medical College and Hospital, Dr MGR Educational and Research Institute, Chennai, IND; 3 Orthopaedics and Trauma, Southport and Ormskirk Hospital NHS Trust, Southport, GBR; 4 Medicine, Shri Madan Lal Khurana Chest Clinic, New Delhi, IND

**Keywords:** patient prioritization, orthopaedic procedures, risk prediction, covid-19, orthopaedics, preparedness index

## Abstract

The coronavirus disease 2019 (COVID-19) pandemic has increased the vulnerability of routine surgical procedures and elective surgery preparedness all over the world, with the suspension of most elective surgeries during the pandemic and the backlog of patients currently on waiting lists, especially in publicly funded healthcare systems. On average, at the beginning of the year 2022, about 200 million patients awaited surgery all over the world. By enhancing the strength of surgical preparedness, there is a better chance of strengthening elective surgical systems against shocks such as future pandemics or climate emergencies. We explore the implications, challenges, and strategies of the concept of surgical preparedness to maintain sustainability in the global healthcare system, especially in low- and middle-income countries (LMICs), with the experiences gained during the COVID-19 pandemic.

## Editorial

A major break in healthcare delivery occurred globally during the outbreak of the coronavirus disease 2019 (COVID-19) pandemic, with the need to implement restrictions and reduce the transmission of severe acute respiratory syndrome coronavirus 2 (SARS-CoV-2) through various government policies. The pandemic brought the vulnerability of routine, elective surgical procedures all over the world to the forefront due to the redeployment of healthcare services on the coronavirus frontlines. Effective vaccination strategies and infection prevention strategies have mitigated some of the resultant fallout; however, elective surgery recovery is still lagging due to the lack of essential surgical preparedness in the event of such global events. On average, at the beginning of the year 2022, about 200 million patients awaited surgery all over the world [1].

For various critical illnesses like cancer, one out of seven patients could not undergo surgery as per plans during the outbreaks of SARS-CoV-2, and various other patients with non-urgent conditions such as osteoarthritis of the hip or knee in need of planned surgical interventions have had prolonged delays in surgical care [1]. Many patients were not even contacted regarding the need for surgery, leading to high rates of disability and the loss of plenty of years of healthy living. Due to the failure to execute elective surgeries on a large scale, there was a high impact on various patients and catastrophic effects on the healthcare system all over the world.

Surgical Preparedness Index (SPI)

The Lancet has published a key indicator of the preparedness of the healthcare systems, the SPI, particularly in the context of elective surgical recovery and COVID-19 [2]. Surgical preparedness is elucidated as the capability of a health organization to maintain and sustain the elective surgical volume while there are pressures from outside systems like pandemics, which are either airborne or non-airborne, warfare, seasonal variations, natural calamities, and political consequences. The National Confidential Enquiry into Patient Outcome and Death (NCEPOD) has classified planned or elective surgery as an intervention planned or booked in advance of routine admission to a hospital under local, regional, or general anesthesia to suit the patient, hospital, and staff.

COVID-19 has had a devastating effect on such patients awaiting elective procedures due to the efforts that were diverted towards tackling the COVID-19 pandemic. Most of the orthopaedic procedures falling into this category, such as total hip or knee arthroplasty, have consequently taken a backseat due to a lack of surgical preparedness in the global healthcare system, with patients awaiting on the surgical waiting lists describing their condition as "worse than death" [3].

Indicators and parameters of SPI

The SPI includes 23 indicators, which are scored from very weak to very strong, and the overall score is between 23 and 115 [1]. The indicators are divided into four categories, viz., various facilities and available consumables (11), adequate staffing (2), prioritizing (2), and available systems (8), as mentioned in Table 1.

**Table 1:** Parameters of SPI SPI: Surgical Preparedness Index

Various facilities and available consumables (11)
1. Ring-fenced theaters. 2. Ring-fenced beds. 3. Ring-fenced critical care. 4. Rearrange areas to give separate paths for elective surgeries. 5. Wide reach to diagnostic and intervention areas. 6. Proper supply of electricity. 7. Proper supply of medications to patients. 8. Proper supply of oxygen. 9. Proper supply of implants and other equipment. 10. Adequate sterilization of hospital instruments. 11. Adequate supply of protection equipment
Adequate staffing (2)
1. Redistribution of staff to areas in need. 2. Ring-fenced teams
Prioritizing (2)
1. Prioritizing patients. 2. Prioritizing procedures
Available systems (8)
1. Proper prior planning for carrying out the operation. 2. Proper assessment preoperatively. 3. Proper testing preoperatively. 4. Facilities to transfer between hospitals when in need. 5. Discharge the patients on time. 6. Support for social well-being. 7. Tele-consultation practices. 8. Adequate means to communicate with the family

The facilities and consumables include ring-fenced operating theaters, hospital beds, critical care beds for planned surgery, flexible hospital areas, diagnostics access, and early identification and treatment of surgical complications, including the maintenance of an adequate supply of electricity, oxygen, drugs, and devices, the availability of sterilization, and personal protective equipment (PPE). The staffing component incorporates the ability to redistribute staff within and between hospitals and ring-fenced teams to provide adequate surgical care. The surgical prioritization concept depends on the ranking of patients according to their medical, surgical, and disability indices. The systems component incorporates formal plans to execute surgeries as planned during emergent situations, preoperative check-ups and testing, adequate resources to transfer the patients to other hospitals, discharge of patients on time, availability of social support, a safety outreach mechanism to monitor the patients from the periphery, and effective communication with the family members. 

Significance of SPI in orthopaedics

There has been a vast restructuring in the healthcare system, with a marked decrease in elective surgeries and outpatient numbers and a massive drop in the number of patients post-COVID-19 pandemic. In the field of elective orthopaedics, patients have noticed a significant deterioration of quality of life (QoL) and function while awaiting pain-relieving procedures.

Various strategies have been considered with predictive models and tools to quantify the backlogs and develop and improve surgical preparedness [3,4]. The development of SPI will help us to delineate the problems and anticipate them beforehand so that we can effectively plan to tackle the situation in our worst times of crisis. The presence of an effective healthcare system, especially hospitals that are well-equipped and have adequate staffing, proper means of transport, an efficient team of doctors comprising trauma surgeons and anesthetists, and sufficient funds, will help us in situations of health crises occurring at the national and global level. By implementing a proper SPI, orthopaedic traumas can be managed alongside elective surgeries, especially tumor surgeries and other orthopaedic emergencies.

Challenges in low- and middle-income countries (LMICs): the 4S approach

The 4S approach comprises the following: (1) structure and facilities (resource development, expansion of facilities); (2) staffing; (3) surgical prioritization; and (4) infrastructure (development and funding) and government involvement [5].

Infection prevention and control

Infections associated with healthcare are relatively uncommon in orthopaedic surgeries and trauma surgeries in comparison to the wards of other surgical branches. At present, the lifetime risk for infection following primary total hip and total knee arthroplasties is about 1%, and the risk level rises to 2-5% after revision surgeries. Infection prevention is of higher importance in orthopaedic surgeries. SPI surveillance every year brings about a proper way to prevent and control infection. In a study conducted by Douglas et al., it was found that there was a 30% fall in infection rates when a multimodality program was implemented, which included a committed team for maintaining hospital hygiene, guidelines elaboration, proper education of the staff personnel, avoiding unnecessary urinary catheterization, active surveillance after discharge from the hospital, and limiting the flow of people into the operating room [6].

Recovery after the COVID-19 pandemic is a challenge. However, the experience of COVID-19 can be utilized to strengthen the global healthcare system with the development of indices such as SPI to enhance resilience and support patient care. In the LMIC healthcare system, the 4S strategies have to be discussed at the national level, including various departments, to implement effective health measures in the face of such natural (global warming) or artificial calamities, which can be expected in the future. This has paved the way for an increase in the number of elective surgeries comparable to the pre-COVID-19 era. Alongside, there's a requirement for a regular (at least yearly) assessment of the hospital using the SPI parameters. By enhancing the strength of preparedness, there is a better chance of strengthening elective surgical procedures against shocks that could occur in the future, and this could help us to upgrade the number of surgeries to meet the increasing demands.
